# Exosomes from Cancer-associated Fibroblasts Orchestrate Lung Metastasis of Breast Cancer via LncRNA LMAEP

**DOI:** 10.7150/jca.139124

**Published:** 2026-09-18

**Authors:** Liyan Lao, Huiping Chen, Huiyi Zeng, Penghan Huang, Yufei Chen, Jianing Chen, Wei Wu, Wenfeng Zeng

**Affiliations:** 1 Guangdong Provincial Key Laboratory of Malignant Tumor Epigenetics and Gene Regulation, Medical Research Center, Sun Yat-Sen Memorial Hospital, Sun Yat-Sen University, Guangzhou 510120, China.; 2 Breast Tumor Center, Sun Yat-Sen Memorial Hospital, Sun Yat-Sen University, Guangzhou 510120, China.; 3 The First Affiliated Hospital, Jinan University, Guangzhou, Guangdong 510632, China.

**Keywords:** breast cancer, lung metastasis, cancer-associated fibroblasts, LncRNA

## Abstract

Lung metastasis is a leading cause of patient mortality in breast cancer. Cancer-associated fibroblasts (CAFs), the most abundant stromal cells within the tumor microenvironment, are closely implicated in pulmonary metastasis. Nevertheless, the mechanism underlying CAF-mediated metastasis remains elusive. We found that density of CAFs in primary tumors showed no significant difference in patients with or without lung metastasis. However, CAFs of breast cancer patients with lung metastasis (termed lung metastasis-associated fibroblasts, LMAFs) exhibited immense competence in inducing epithelial-mesenchymal transition (EMT), invasion and proliferation of tumor cells relative to non-metastatic CAFs. LMAF-derived exosomes were sufficient to confer the pro-metastatic phenotype to tumor cells. LncRNA microarray screening identified LMAEP (Lung Metastasis-Associated EMT Promoter) as a lncRNA specifically enriched in LMAF-derived exosomes. Knockdown of LMAEP abrogated the capacity of LMAFs to induce cancer cell EMT, invasion and proliferation. Targeting of lncRNA LMAEP tremendously suppressed tumor growth and lung metastasis of breast cancer* in vivo*. These findings identify LMAEP as a critical metastasis-inducing mediator of CAFs, positioning it as a potential biomarker for risk prediction and early detection of lung metastasis. Furthermore, targeting exosomal lncRNA LMAEP represents a promising therapeutic strategy for restraining pulmonary metastasis of breast cancer.

## Introduction

Breast cancer ranks as the most prevalent malignant tumor among females worldwide. Despite the continuous advancement of early screening protocols and multimodal therapeutic strategies, distant metastasis remains the primary culprit for treatment failure and poor long-term survival in affected patients 1, 2. Among the various metastatic manifestations, lung metastasis represents one of the most common distant metastatic sites of breast cancer, characterized by high incidence and dismal prognosis, thus emerging as a critical challenge that urgently demands resolution in the field of breast cancer clinical translational research 3, 4. Tumor metastasis is not an autonomous process of cancer cells, but rather a sophisticated, multi-step biological cascade orchestrated by the synergistic interplay between cancer cells and the surrounding tumor microenvironment (TME). The dynamic bidirectional crosstalk between stromal cells and cancer cells exerts a decisive role in regulating cancer cell epithelial-mesenchymal transition (EMT), invasive migration, and subsequent colonization at distant organs.

Cancer-associated fibroblasts (CAFs) are the most abundant and functionally pivotal stromal cell population within the TME. Distinguished from normal fibroblasts, activated CAFs exhibit characteristic morphological changes (spindle-shaped morphology), enhanced proliferative capacity, and robust secretory activity. They can comprehensively modulate tumor progression through multiple mechanisms, including the secretion of cytokines and growth factors, remodeling of the extracellular matrix, and release of exosomes 5, 6. CAFs display pronounced functional heterogeneity, with specific subsets demonstrated to potentiate breast cancer lung metastasis 7. However, the precise molecular mechanisms underlying CAF-mediated promotion of breast cancer lung metastasis remain incompletely defined, and the key functional components responsible for exerting pro-metastatic effects via paracrine pathways await further precise identification and validation.

Exosomes are nano-scale lipid vesicles actively secreted by cells, which can stably encapsulate a diverse array of bioactive molecules, such as proteins, messenger RNAs (mRNAs), microRNAs (miRNAs), and long non-coding RNAs (lncRNAs). Serving as core mediators of intercellular communication within the TME, exosomes enable the long-distance transmission and functional modulation of signaling molecules between cells. In recent years, lncRNAs have garnered substantial attention in tumor metastasis research due to their tissue-specific expression patterns and diverse regulatory pathways. Exosome-derived lncRNAs can reprogram cancer cell phenotypes and augment their metastatic potential through mechanisms including competing endogenous RNA (ceRNA) regulation, chromatin modification, and signaling pathway activation. Currently, research investigating the regulatory role of CAF-derived exosomal lncRNAs in breast cancer lung metastasis is still in its infancy. In particular, there is a paucity of targeted studies focusing on lung metastasis-specific CAF subsets, and the association between CAF heterogeneity and exosomal lncRNA expression profiles, as well as the core regulatory function of specific lncRNAs in breast cancer lung metastasis, have not yet been clearly elucidated.

Based on the aforementioned research background and existing gaps in the field, the present study is clinically oriented towards addressing the challenge of breast cancer lung metastasis. By focusing on the functional heterogeneity of CAFs, we aim to identify and characterize lung metastasis-associated fibroblasts (LMAFs), and investigate the regulatory effects of their secreted exosomes on breast cancer cell invasion and EMT. LncRNA microarray analysis will be employed to screen for key lncRNAs specifically enriched in LMAF-derived exosomes. Furthermore, the inhibitory efficacy of targeting this lncRNA on breast cancer lung metastasis will be validated both at the cellular level and in a cell co-implantation model using immunodeficient mice. This study aims to elucidate a novel mechanism by which CAFs regulate breast cancer lung metastasis via exosomal lncRNAs, thereby providing potential molecular therapeutic targets and intervention strategies for breast cancer lung metastasis. In doing so, it seeks to address the current research deficiencies regarding CAF heterogeneity and exosomal non-coding RNA-mediated regulation in this field.

## Method and Materials

### Participants and human tumor samples

Breast tumor samples were obtained from 60 female individuals aged 30-55 years with invasive breast carcinoma at the Sun Yat-Sen Memorial Hospital, Sun Yat-Sen University, between 2020 and 2025 and were used for immunostaining. Fresh tumor samples obtained from another cohort of female individuals with breast cancer aged 30-55 years at the Sun Yat-Sen Memorial Hospital, Sun Yat-Sen University, between 2020 and 2021 were used for primary CAF isolation. All samples were collected with informed consent from participants, and all related procedures were performed with the approval of the internal review and ethics boards of Sun Yat-Sen Memorial Hospital.

### Cell culture

Human breast cancer cell lines MDA-MB-231 (RRID: CVCL_0062) and MCF-7 (RRID: CVCL_0031) were obtained from ATCC and maintained in DMEM supplemented with 10% FBS and 1% penicillin/streptomycin. The cell lines were tested negative for mycoplasma contamination. Primary CAFs (CTRL-CAFs and LMAFs) were isolated from clinical breast cancer specimens as previously described 8. For stable knockdown of LMAEP, LMAFs were transduced with lentiviral vectors expressing shRNAs targeting LMAEP (sh-LMAEP-1: 5'-GGGCTGTGAAGCTCTACAACAGCTTCAAGAGAGCTGTTGTAGAGCTTCACAGCCCTTTTTT-3'; sh-LMAEP-2: 5'-GGCCTTGTCTGTTGAGAAAGACGTTCAAGAGACGTCTTTCTCAACAGACAAGGCCTTTTTT-3') or a control shRNA (shGFP: 5'-GCAAGCTGACCCTGAAGTTCATTCAAGAGATGAACTTCAGGGTCAGCTTGCTTTTTT-3'). Transduced cells were selected using puromycin (2 μg /mL) and cultured in media with exosome-depleted FBS for further experiments.

### Isolation and characterization of extracellular vesicles

Isolation of CAF-derived extracellular components was performed via differential ultracentrifugation as previously described 8. Briefly, CAFs were cultured in DMEM supplemented with 10% exosome-depleted FBS (Gibco). Upon reaching 80% confluence, the conditioned media (CM) were harvested and subjected to a series of centrifugation steps at 4 °C to remove cellular debris and large vesicles. The CM was first centrifuged at 10000 g for 20 min. The resulting pellet was collected as fraction C1, which contains larger vesicles and cellular debris. To isolate exosomes, the remaining supernatant was passed through a 0.22 μm filter (Millipore) and subsequently ultracentrifuged at 200,000 g for 80 min. The resulting pellet, containing the exosome-enriched fraction (C2), was washed once with ice-cold PBS and ultracentrifuged again at the same speed to ensure purity. The final supernatant, designated as the non-vesicular soluble fraction (C3), was collected for downstream analysis. All protein concentrations were quantified using a BCA assay (Pierce), and the isolated vesicles were stored at -80 °C or used immediately for functional assays. Exosome morphology was visualized by Transmission Electron Microscopy (TEM), and size distribution was determined by Nanoparticle Tracking Analysis (NTA) using NanoSight NS300. Protein markers (CD63, CD81, and ALIX) were validated by Western blotting.

### Immuno-magnetic isolation of CD81^+^ exosomes

To obtain high-purity exosome subpopulations, CD81^+^ exosomes were isolated from the pre-cleared conditioned media (CM) using the Exosome Isolation Kit (Miltenyi Biotec, Cat#130-110-914) according to the manufacturer's instructions. Briefly, the CM was first subjected to sequential centrifugation to remove cells and large debris. The resulting supernatant was incubated with Exosome Isolation MicroBeads for 1 hour at room temperature with gentle agitation, allowing the magnetic beads to specifically bind to the CD81 antigen on the exosome surface. For magnetic separation, a μColumn was placed in the magnetic field of a MACS Separator and equilibrated with 100 μL of Isolation Buffer. The bead-exosome suspension was then loaded onto the column. The column was washed three times with 200 μL of Isolation Buffer to remove non-specific background and unlabelled vesicles. Subsequently, the column was removed from the magnetic field, and the magnetically labeled CD81^+^ exosomes were eluted with 100 μL of Isolation Buffer. The purified exosomes were immediately used for downstream functional assays or stored at -80 °C.

### RNA Fluorescence *In Situ* Hybridization (RNA-FISH)

To determine the subcellular localization and frequency of LMAEP^+^ cells, RNA-FISH was performed using Alexa Fluor 488-labeled probes specific to LMAEP. Cells were fixed in 4% paraformaldehyde and hybridized overnight at 37 °C following the manufacturer's instructions. Images were captured using a confocal microscope, and the proportion of LMAEP^+^ CAFs was quantified from five independent fields per group.

### Quantitative RT-PCR

Total RNA from cells or isolated exosomes was extracted using Trizol reagent. For exosomal RNA, cel-miR-39 was added as a spike-in control. cDNA was synthesized and qRT-PCR was performed using SYBR Green Master Mix. Data were collected and analyzed using a LightCycler 480 instrument (Roche). Relative expression levels of LMAEP were calculated using the 2^-Delta/Delta Ct method, with GAPDH or spike-in as internal controls.

### Transwell invasion assay

Cell invasion capacity was evaluated using 24-well Transwell chambers with 8-μm pore size inserts (Corning, NY, USA) pre-coated with Matrigel. Briefly, the upper surfaces of the Transwell membranes were coated with 50 μL of growth factor-reduced Matrigel (BD Biosciences; diluted 1:8 in serum-free medium) and incubated at 37 °C for 2 hours to allow for basement membrane reconstruction. Following stable transduction or treatment, MDA-MB-231 or MCF-7 cells were starved in serum-free medium for 12 hours. Subsequently, 5 × 10^4^ cells suspended in 200 μL of serum-free DMEM were seeded into the upper chamber. To assess the pro-invasive effect of LMAFs or their derived exosomes, the lower chamber was filled with 600 μL of conditioned medium (CM) or medium supplemented with isolated exosomes (50 μg/mL). Medium containing 10% exosome-depleted FBS served as a chemoattractant. For cargo-depletion experiments involving enzymatic treatment, exosomes were permeabilized and incubated with nuclease 1 (RNase A, 100 U/mL) or nuclease 2 (RNase I, 100 U/mL), or protease (proteinase K, 100 μg/mL) for 30 min at 37 °C before being added to the lower chamber.

### Immunoblot

Total protein was extracted from cell lysates or isolated exosomes using RIPA buffer (Thermo Fisher Scientific) supplemented with 1% protease and phosphatase inhibitor cocktails (Roche). For exosomal protein preparation, the pellet was directly lysed in a minimized volume of RIPA buffer and quantified using a BCA Protein Assay Kit (Pierce, IL, USA). Equal amounts of protein (20-40 μg per lane) were denatured at 95 °C for 10 min, separated by 10% or 12% SDS-PAGE, and subsequently transferred onto PVDF membranes (Millipore, MA, USA). The membranes were blocked with 5% non-fat milk or bovine serum albumin (BSA) in TBST for 1 hour at room temperature. Following blocking, the membranes were incubated overnight at 4 °C with primary antibodies against: CD63 (Novus, Cat# NBP2-42225, 1:1000), ALIX (Novus, Cat# NBP1-90201, 1:1000), CD81 (Novus, Cat#NBP2-53318, 1:1000), Vimentin (Cell Signaling Technology, Cat#5741T, 1:1000), E-cadherin (Cell Signaling Technology, Cat#3195T, 1:1000), and GAPDH (HRP-60004, Proteintech; 1: 10,000). After washing three times with TBST, the membranes were incubated with HRP-conjugated secondary antibodies (1:5000) for 1 hour. Protein bands were visualized using an Enhanced Chemiluminescence (ECL) Detection System (Bio-Rad, CA, USA).

### Immunofluorescence and digital image analysis

Immunofluorescence (IF) staining was performed on 4-μm thick paraffin-embedded sections or fixed cell slides. Briefly, sections were deparaffinized, rehydrated, and subjected to antigen retrieval in EDTA (pH 9.0) using a microwave. Following blocking with 5% goat serum for 1 hour, sections were subjected to RNA-FISH for LMAEP and then incubated with primary antibody against α-SMA (Cell Signaling Technology, Cat#19245T, 1:200), overnight at 4 °C. Appropriate Alexa Fluor-conjugated secondary antibodies (1:500, Thermo Fisher Scientific) were applied for 1 hour, and nuclei were counterstained with DAPI. Full-slide fluorescence images were acquired using the KFBIO KF-FL-005 Digital Slide Scanner (KFBIO, Ningbo, China) at 20 × magnification. To ensure image consistency, all slides within the same experiment were scanned using identical exposure times and gain settings. Quantitative analysis of the digital slides was performed using HALO software (Indica Labs, Albuquerque, NM, USA). The Highplex FL module was utilized for multi-channel cell segmentation and phenotyping. Specifically, DAPI signal was used for nuclear segmentation, while cytoplasmic markers (α-SMA for CAFs) defined the cellular boundaries. The spatial distribution of α-SMA^+^ CAFs relative to tumor nests were automatically calculated across the entire tissue section. At least three representative regions of interest (ROIs) or the entire tumor area were analyzed per sample.

### EdU incorporation assay

To assess tumor cell proliferation, MCF-7 or MDA-MB-231 cells were treated with indicated conditioned media or exosomes. Cells were then incubated with 10 μM EdU (5-ethynyl-2′-deoxyuridine) for 2 hours at 37 °C. After EdU labeling, cells were harvested, washed with PBS, and fixed with 4% paraformaldehyde for 15 minutes at room temperature. Cells were then permeabilized with 0.5% Triton X-100 in PBS for 10 minutes, followed by washing with 3% BSA in PBS. Click reaction was performed by incubating cells with reaction cocktail containing CuSO₄, Alexa Fluor-488 azide, and reducing agent according to the manufacturer's instructions (EdU Cell Proliferation Kit, Thermo Fisher, C10418) for 30 minutes at room temperature, protected from light. After washing, cells were resuspended in PBS and analyzed using a CytoFLEX flow cytometer (Beckman Coulter). The proportion of EdU-positive cells was quantified using FlowJo software.

### Enzyme-Linked Immunosorbent Assay (ELISA)

Conditioned media from primary tumor cultures were collected and centrifuged to remove debris. Levels of metastasis-associated cytokines were measured using commercially available ELISA kits according to the manufacturer's instructions: CCL18 (Invitrogen, EHCCL18), IL-6 (eBioscience, 88-7066-86), IL-8 (eBioscience, 88-8086-22), IL-10 (eBioscience, 88-7106-22), TNF-α (eBioscience, 88-7346-22), TGF-β1 (R&D Systems, DB100B), VEGF (R&D Systems, DVE00) and CXCL12 (R&D Systems, DSA00). Absorbance was read at 450 nm using a microplate reader (Bio-Tek), and cytokine concentrations were calculated from standard curves.

### Immunohistochemistry

For immunohistochemical analysis of proliferation, primary tumor tissues were fixed in 4% paraformaldehyde, embedded in paraffin, and sectioned at 4-μm thickness. Sections were subjected to antigen retrieval in citrate buffer (pH 6.0) and incubated with anti-Ki67 antibody (Starter, S0B2332, 1:500) overnight at 4 °C, followed by HRP-conjugated secondary antibody and DAB substrate. Images were acquired using a light microscope (Olympus), and the percentage of Ki67-positive cells was quantified in at least five random fields per section using ImageJ software.

### Orthotopic xenograft and IVIS imaging

All mice were bred in the specific pathogen-free animal facility of the Laboratory Animal Resource Center of Sun Yat-Sen University in individually ventilated cages. All animal procedures were approved by the Institutional Animal Care and Use Committee of Sun Yat-sen University. Female NSG mice (6-8 weeks old) were randomly assigned to four groups. MDA-MB-231 cells (1 × 10^6^) were co-injected with CTRL-CAFs, sh-LMAEP-LMAFs, or shGFP-LMAFs in a 1:3 ratio into the fourth mammary fat pad. Tumor growth was monitored by caliper measurements and In Vivo Imaging System (IVIS). Bioluminescence was quantified as total photon flux (photons/s) within defined ROIs using Living Image software. For evaluation of lung metastasis, lungs were harvested, fixed in 4% paraformaldehyde, embedded in paraffin, sectioned at 4-μm thickness, and stained with hematoxylin and eosin (H&E). Metastatic foci were counted under a light microscope by two independent investigators blinded to the experimental groups. The number of pulmonary metastases per 1 cm² of lung tissue was recorded for each mouse. The metastatic index was calculated as the number of lung metastatic foci divided by the volume of the primary tumor (10² mm^3^) for each mouse. Tumor volume was estimated using the formula V = (L × W² × π) / 6, where L and W represent the longest and shortest diameters of the primary tumor, respectively.

### Statistical analysis

Data are presented as mean ± s.d. from at least three independent experiments, except for the tumor growth curves in Fig. 5A, which are presented as mean ± s.e.m. Statistical significance was evaluated using unpaired Student's t-test for two-group comparisons or one-way ANOVA test for multiple comparisons. p < 0.05 was considered statistically significant.

## Results

### CAFs of lung-metastatic patients possessed robust pro-invasive and pro-proliferative potential

Accumulating evidence has established a close correlation between activated fibroblasts in tumor microenvironment and malignant tumor metastasis 9, 10. In this study, we collected paraffin-embedded tissue sections of primary breast cancer from 60 patients with or without lung metastasis and quantified the density of α-smooth muscle actin (α-SMA)-positive cells, indicating activated fibroblasts in tumor microenvironment, via immunofluorescence staining (Fig. 1A). Notably, there was no significant difference in the density of α-SMA-positive cells across breast cancer subtypes with distinct pathological classifications (Fig. 1A, B). Surprisingly, the primary tumors of patients with lung metastasis did not show higher abundance of α-SMA-positive cells than those without pulmonary involvement (Fig. 1C). These findings suggest that the mere number of activated CAFs is not a decisive determinant governing the metastatic potential of malignant tumor cells.

CAFs are well known for their pronounced functional heterogeneity 11, 12. Previous studies demonstrated that secretions from some CAF subsets displayed evident capacity to precipitate the development of lung metastasis of malignancies 13, 14. To assess the functional significance of CAFs, primary tumor specimens were collected from breast cancer patients with or without lung metastasis. Primary CAFs were isolated from the tumor samples, cultured *in vitro*, and the conditioned media (CM) were harvested. MCF-7 and MDA-MB-231 breast cancer cell lines were then exposed to conditioned media of CAFs from different patients, and their invasive capacity was evaluated using transwell invasion assays. We selected MCF-7 (luminal A, epithelial, low baseline invasiveness) and MDA-MB-231 (triple-negative, invasive) cells to represent distinct breast cancer subtypes, enabling assessment of invasion enhancement across low- and high-baseline invasive background. Evidence showed that conditioned media from CAFs of non-metastatic breast cancer patients exerted no significant effect over the breast cancer cells with different invasive potential (Fig. 1D, E). In stark contrast, conditioned media derived from CAFs of patients with breast cancer lung metastasis significantly promoted cancer cell invasion, albeit with considerable heterogeneity across individual isolates (Fig. 1D, E). Epithelial-mesenchymal transition (EMT) is widely recognized as a hallmark event that augments the invasive and metastatic capabilities of tumor cells 15, 16. We therefore evaluated the EMT status of MCF-7 cells following treatment with conditioned media from different CAFs. Consistently, CAFs from patients with lung metastasis robustly induced EMT in breast cancer cells, manifested by reduced E-cadherin expression and elevated vimentin levels, again exhibiting marked individual variability (Fig. 1F). We next examined whether CAFs from patients with lung metastasis affect tumor cell proliferation. EdU incorporation assay revealed that conditioned media from these CAFs, but not from non-metastatic CAFs, significantly increased the proportion of EdU-positive tumor cells (Fig. 1G), indicating that lung-metastatic CAFs possessed pro-proliferative capacity in addition to their pro-invasive and pro-EMT effects. We hereby designated this subset of CAFs, derived from primary tumors of breast cancer patients with lung metastasis and capable of initiating cancer cell EMT, invasion and proliferation, as lung metastasis-associated fibroblasts (LMAFs).

### LMAFs drove breast cancer EMT, invasion and proliferation through exosomes

To pinpoint the components within LMAF-conditioned media responsible for tumor invasion and EMT, we fractionated the conditioned media using differential centrifugation 17. Breast cancer cells were then exposed to distinct fractions of LMAF-conditioned media (C1, C2, C3). Notably, fraction C2 of LMAF-conditioned media, but not fractions C1 or C3, exhibited potent pro-invasive activity over MCF-7 and MDA-MB-231 cells (Fig. 2A) and induced vimentin upregulation alongside E-cadherin downregulation in MCF-7 cells (Fig. 2B), recapitulating the activity of the entire conditioned media. Moreover, transmission electron microscopy (TEM) revealed that the pellets of C2 fraction displayed a typical cup-shaped structure (Fig. 2C), a morphological feature characteristic of exosomes. Nanoparticle tracking analysis (NTA) demonstrated a size distribution centered around 100 nm (Fig. 2D). Western blotting further confirmed that the C2 fraction expressed the established exosomal markers Alix, CD81, and CD63 (Fig. 2E). These findings indicated that the C2 fraction contains abundant exosomal components. Importantly, depletion of exosomes in the conditioned media of LMAFs using immunomagnetic beads resulted in a significant abrogation of the ability of LMAF-conditioned media to enhance invasive capacity and EMT of breast cancer cells (Fig. 2F, G). Consistent with the pro-invasive and pro-EMT effects, EdU incorporation assay revealed that depletion of exosomes from LMAF-CM abrogated its pro-proliferative effect (Fig. 2H), confirming that exosomes are the key carriers mediating LMAF-induced tumor cell proliferation. Collectively, these findings demonstrate that LMAFs promote breast cancer cell invasion, EMT and proliferation through exosomes.

### LMAEP emerged as a signature lncRNA in pro-metastatic exosomes of LMAFs

Serving as indispensable mediators of paracrine and long-distance intercellular communication, exosomes orchestrate these processes via their encapsulated proteins and nucleic acids, which function as core regulatory molecules 18. To identify the components within LMAF-derived exosomes responsible for tumor cell invasion, EMT and proliferation, the isolated exosomes were pretreated with proteinase K or nucleases, respectively, prior to coculturing with breast cancer cells to deplete protein or nucleic acid components. We found that the pretreatment of nucleases, instead of proteinase, substantially attenuated the ability of LMAF-derived exosomes to drive breast cancer cell invasion and EMT (Fig. 3A, B), pointing to a central role of nucleic acids in LMAF exosome-mediated invasion.

Exosomes are known to package long non-coding RNAs (lncRNAs) and deliver them as paracrine regulatory signals 19. To identify candidate lncRNAs responsible for the pro-metastatic effects of LMAFs, we isolated exosomes from LMAFs or CAFs from non-metastatic patients and performed lncRNA microarray profiling. LncRNA microarray profiling revealed remarkable elevation of multiple lncRNAs in LMAF-derived exosomes compared with those from non-metastatic CAFs (Fig. 3C, D). Among the differentially expressed transcripts, lnc-NT5C3B-3:1 exhibited the highest fold-change. Quantitative real-time polymerase chain reaction (qRT-PCR) of CAF-derived exosomes from independent patient samples confirmed that lnc-NT5C3B-3:1 was the most significantly elevated lncRNA in LMAF-derived exosomes, with consistent upregulation across biological replicates, whereas the other candidates exhibited either no statistically significant upregulation or highly heterogeneous expression across samples (Fig. 3E). Consistent with the exosome data, lnc-NT5C3B-3:1 expression was also elevated in LMAFs compared with non-metastatic CAFs (Fig. 3F). Moreover, in situ hybridization assays revealed the existence of lnc-NT5C3B-3:1 in the cytoplasm of LMAFs and higher abundance of it in LMAFs versus non-metastatic CAFs (Fig. 3G, H). Therefore, we prioritized this lncRNA for functional investigation and designated it LMAEP (Lung Metastasis-Associated EMT Promoter).

### LMAEP knockdown abrogated LMAF-induced tumor cell invasion and proliferation

To evaluate the functional significance of lncRNA LMAEP, we knocked down its expression in LMAFs through shRNAs. qRT-PCR and in situ hybridization assays confirmed the successful silencing of lncRNA LMAEP in LMAFs (Fig. 4A, B). Consistently, the level of lncRNA LMAEP in exosomes derived from LMAFs was sharply reduced (Fig. 4C). Impressively, knockdown of lncRNA LMAEP resulted in a profound reversal of LMAF-induced invasion and mesenchymal transition of breast cancer cells (Fig. 4D, E). In addition to invasion and EMT, we assessed the effect of LMAEP knockdown on tumor cell proliferation. EdU incorporation assay revealed that exosomes from LMAFs significantly increased the proportion of EdU-positive breast cancer cells, whereas exosomes from LMAEP-knockdown LMAFs exhibited a markedly diminished pro-proliferative effect (Fig. 4F). These findings indicated that exosomal lncRNA LMAEP was responsible for LMAF-mediated tumor cell invasion, EMT and proliferation, that targeting LMAEP represented an attractive strategy to abrogate the multifaceted pro-metastatic effects of LMAFs on cancer cells.

Given that LMAEP is required for LMAF-induced tumor cell invasion and proliferation, we next sought to explore the upstream signals that induce LMAEP expression in CAFs. We treated normal fibroblasts with conditioned media (CM) derived from primary tumors of breast cancer patients with or without lung metastasis. Notably, only CM from lung-metastatic tumors significantly induced LMAEP expression in fibroblast-derived exosomes (Fig. 4G). To identify the potential factor responsible for this induction, we performed ELISA screening for several metastasis-associated cytokines in the tumor CM, including IL-6, IL-8, CCL18, TGF-β, TNF-α, IL-10, VEGF and CXCL12^20^-^24^. CCL18, IL-6 and TGF-β were elevated in CM of lung-metastatic tumors compared with non-metastatic CM, while other tested factors exhibited no significant differences between the two groups (Fig. 4H). Treatment of normal fibroblasts with recombinant CCL18, but not IL-6 or TGF-β, was sufficient to upregulate LMAEP in their exosomes (Fig. 4I), suggesting that tumor-derived CCL18 may serve as an upstream inducer of LMAEP in CAFs.

### Targeting LMAEP suppressed LMAF-driven lung metastasis* in vivo*

To investigate the *in vivo* efficacy of lncRNA LMAEP targeting, MDA-MB-231 breast cancer cells were co-implanted with different CAFs into the mammary fat pads of immunodeficient mice. We found that tumors in the mice with LMAF co-inoculation exhibited significantly accelerated growth (Fig. 5A), ultimately developing pronounced lung metastasis, as determined by bioluminescence monitoring and histological analysis (Fig. 5B-E). In contrast, silencing lncRNA LMAEP in the co-implanted LMAFs drastically attenuated tumor growth and led to a marked decrease in lung metastasis burden (Fig. 5B-E). Consistent with the *in vitro* findings, immunohistochemical analysis of Ki67 on primary tumor sections revealed that tumors co-injected with LMAFs exhibited substantially higher proliferation rates than those with non-metastatic CAFs, and this enhancement was reversed when LMAEP was silenced (Fig. 5F, G). To determine whether the reduced lung metastasis upon LMAEP knockdown was simply a consequence of smaller primary tumors, we calculated a metastatic index (number of lung metastatic foci per 10² mm^3^ of primary tumor) for each mouse 25. After normalization to primary tumor volume, LMAEP knockdown still significantly reduced metastatic burden compared with the shGFP control (Fig. 5H), indicating a metastasis-specific effect beyond the difference in primary tumor growth.

Collectively, the *in vivo* experimental results established that targeting LMAEP effectively abrogated LMAF-driven breast cancer lung metastasis, highlighting its potential as a therapeutic target for further evaluation.

## Discussion

This study addresses a critical unmet clinical need: the lack of reliable biomarkers and therapeutic targets for breast cancer lung metastasis. We overturn the traditional paradigm by showing that functional heterogeneity of CAFs, rather than their abundance, determines metastatic propensity. More importantly, we identify LMAF-derived exosomal lncRNA LMAEP as a clinically actionable molecule that serves both as a potential biomarker to identify patients at high risk of lung metastasis and a therapeutic target whose inhibition suppresses lung metastasis *in vivo*.

With the in-depth exploration of the tumor microenvironment in recent years, the classification and functional differentiation of CAF subtypes are becoming a research hotspot. CAFs of different origins and activation states exhibit distinct secretion profiles, signaling regulatory pathways, and biological effects on cancer cells 26, 27. Through *in vitro* functional experiments, this study demonstrated that CAFs derived from breast cancer display remarkable heterogeneity in their pro-invasive capacity, with only the LMAF subtype possessing potent pro-metastatic potential. Selectively targeting functionally pathogenic CAF subtypes, as opposed to broad-spectrum CAF intervention, would help circumvent the off-target effects and side effects thereby improving the specificity and safety of treatment. In contrast, broad-spectrum CAF depletion carries well-documented risks, including impaired wound healing, immune disruption, and normal fibroblast mis-targeting 28-30.

Herein, we identified exosomes as the key carriers for CAFs to exert paracrine regulation, through which CAFs deliver functional lncRNAs to orchestrate pro-metastatic effects. While previous studies have established that CAFs primarily govern tumor metastasis by secreting soluble protein factors such as TGF-β, IL-6, and VEGF 31, we uncovered the key role of exosome-derived non-coding RNAs. We demonstrated that LMAF-derived exosomes confer pro-metastatic phenotypes on breast cancer cells, indicating that exosomal cargo is functionally transferred to recipient tumor cells. EMT represents a cardinal event that equips cancer cells with migratory and invasive competence 32, as evidenced by the downregulation of the epithelial marker E-cadherin and the upregulation of mesenchymal markers including vimentin. This EMT-driving capacity elucidates the core biological mechanism underlying the pro-metastatic function of LMAF-derived exosomes. In line with emerging insights in this field 33-35, exosomal lncRNAs are known to regulate EMT-related signaling pathways through diverse mechanisms, including acting as competing endogenous RNA (ceRNA) sponges to sequester tumor-suppressive miRNAs, and modulating classic metastatic pathways including TGF-β/Smad and Wnt/β-catenin. The LMAF-derived exosomal lncRNA LMAEP identified in this study likely operates through analogous pathways, charting a clear course for subsequent mechanistic dissection.

Through lncRNA microarray screening, this study identified and prioritized a specific lncRNA that is selectively enriched in LMAF-derived exosomes. At the cellular level, intervention targeting LMAEP effectively reversed the pro-invasive and pro-proliferative effects driven by LMAF-derived exosomes. *In vivo* animal experiments further confirmed that targeting LMAEP markedly reduced the number and volume of breast cancer lung metastatic foci, firmly establishing its feasibility and efficacy as an anti-metastatic target. Compared with traditional protein targets, exosomal lncRNAs exhibit stronger tumor specificity, and exosomes themselves can serve as natural delivery vehicles, which confers potential advantages for clinical translation. Notably, lncRNA LMAEP is specifically enriched in LMAF-derived exosomes but shows extremely low expression in normal fibroblasts and non-metastatic CAFs, a distribution pattern that may mitigate off-target effects including normal tissue toxicity. This unique profile endows it with dual value as both a diagnostic biomarker and a therapeutic target. In light of recent advances in clinical translational research, specific lncRNAs detected in peripheral blood exosomes have emerged as biopsy-free diagnostic indicators for tumor metastasis. Therefore, LMAEP also offers a blood-based approach to identify breast cancer patients at high risk of developing lung metastasis, which would be particularly valuable for early stage patients for whom current imaging or circulating tumour DNA methods have limited sensitivity. As such, beyond its therapeutic potential, the measurement of circulating exosomal LMAEP holds promise as a clinical biomarker to stratify patients, monitor metastatic risk, and ultimately bridge the fundamental discovery to patient benefit.

Several limitations of this study should be acknowledged. First, the precise molecular target of LMAEP and its downstream signaling pathways remain to be defined. Second, our lncRNA microarray revealed multiple upregulated candidates in LMAF-derived exosomes. Although LMAEP showed the highest and most reproducible fold-change, we cannot exclude the possibility that other lncRNAs also contribute to the pro-metastatic phenotype. Third, the clinical utility of LMAEP as a biomarker and therapeutic target will require validation in larger patient cohorts and advanced preclinical models. Despite these limitations, our findings provide a foundation for future investigation into LMAEP as a promising node for intervention in breast cancer lung metastasis.

## Conclusions

This study establishes functional heterogeneity of CAFs as a pivotal driver in breast cancer lung metastasis, uncovers a novel mechanism by which LMAFs promote breast cancer cell EMT and lung metastasis through exosome-mediated delivery of lncRNA LMAEP, and validates the anti-metastatic efficacy of targeting LMAEP. Collectively, these findings enrich the theoretical framework of stromal-cancer cell crosstalk in the tumor microenvironment and provide a novel target for precision-targeted therapy against breast cancer lung metastasis. It lays a foundation for the subsequent development of exosomal lncRNA-targeted drugs and blood-based diagnostic strategies, underscoring its dual significance in fundamental research and clinical translation.

## Figures and Tables

**Figure 1:**
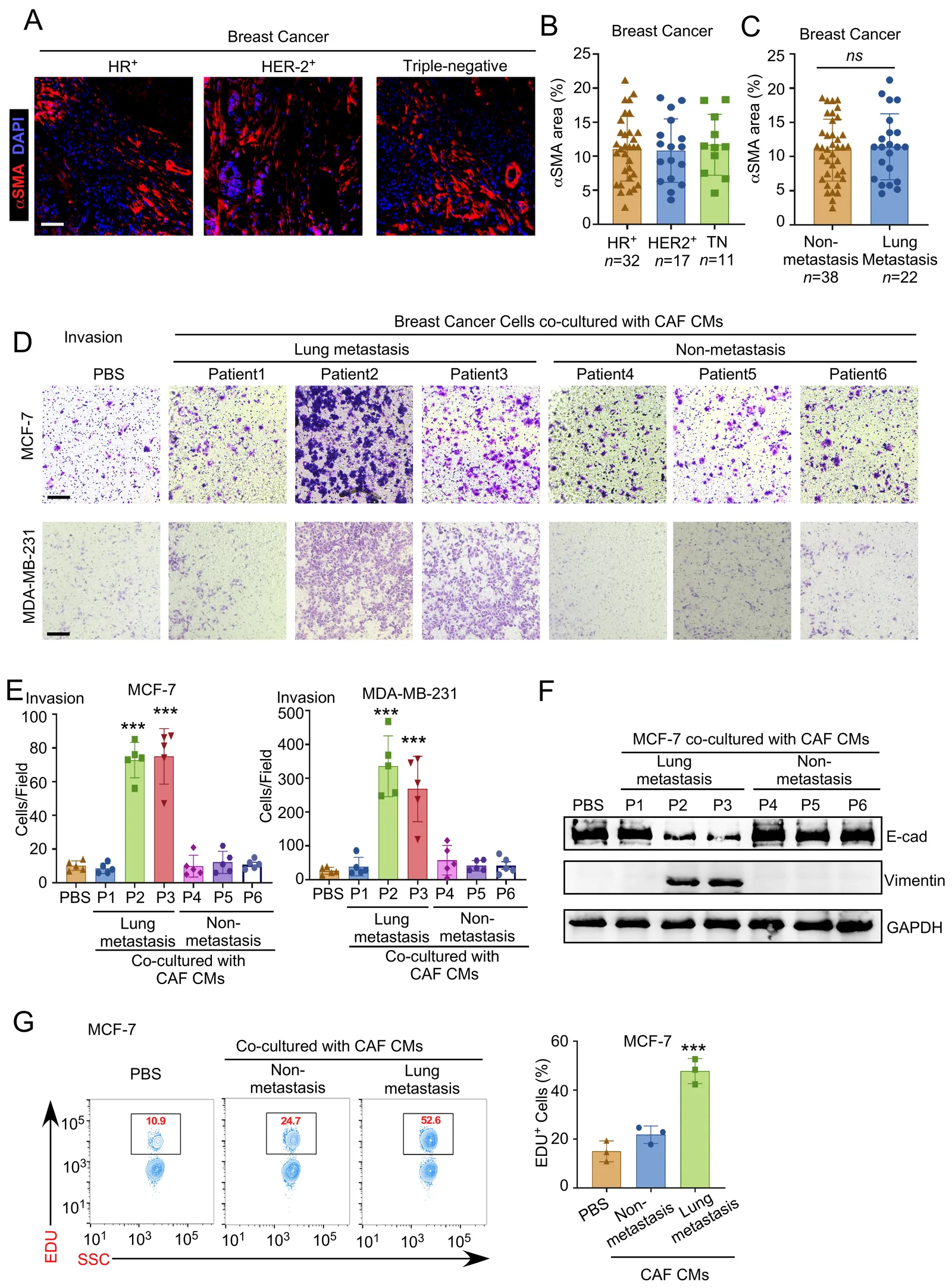
** CAFs of lung-metastatic patients possessed robust pro-invasive and pro-proliferative potential.** A and B. Representative immunofluorescent images (A) and quantification (B) of alpha smooth muscle actin (α-SMA) staining in breast cancer (BC) tissues of different molecular subtypes. HR⁺ (Hormone receptor-positive, *n* = 32), HER2⁺ (HER2-positive, *n* = 17) and TN (Triple-negative, *n* = 11). C. Quantification of α-SMA immunostaining in BC patients with or without lung metastasis (lung metastasis, *n* = 38; non-metastasis, *n* = 22). *ns*, not significant. D and E. Representative images (D) and quantification (E) of the Transwell invasion assay showing the invasive capacity of breast cancer cells through a Matrigel-coated membrane. Briefly, MCF-7 and MDA-MB-231 breast cancer cell lines were treated with CAF conditioned media (CM) derived from indicated breast cancer sample. Following a 48-hour incubation toward a chemoattractant gradient, cells that penetrated the matrix were fixed, stained with crystal violet, and quantified across multiple random fields. MCF-7 and MDA-MB-231 cells treated with PBS were set as control. P1, patient 1. F. Representative immunoblotting for E-cadherin (E-cad) and Vimentin of MCF-7 treated with indicated CAF CMs (*n* = 3 independent experiments). G. EdU incorporation assay in MCF-7 tumor cells treated with CM of CAFs from patients with or without lung metastasis. PBS-treated cells served as control (*n* = 3 independent experiments). Data are presented as mean ± s.d. *** p < 0.001 by one-way ANOVA with Dunnett's test compared to PBS group in (E, G), *p* values by unpaired Student's t-test in (C), and *p* values by one-way ANOVA with Tukey's test in (B).

**Figure 2:**
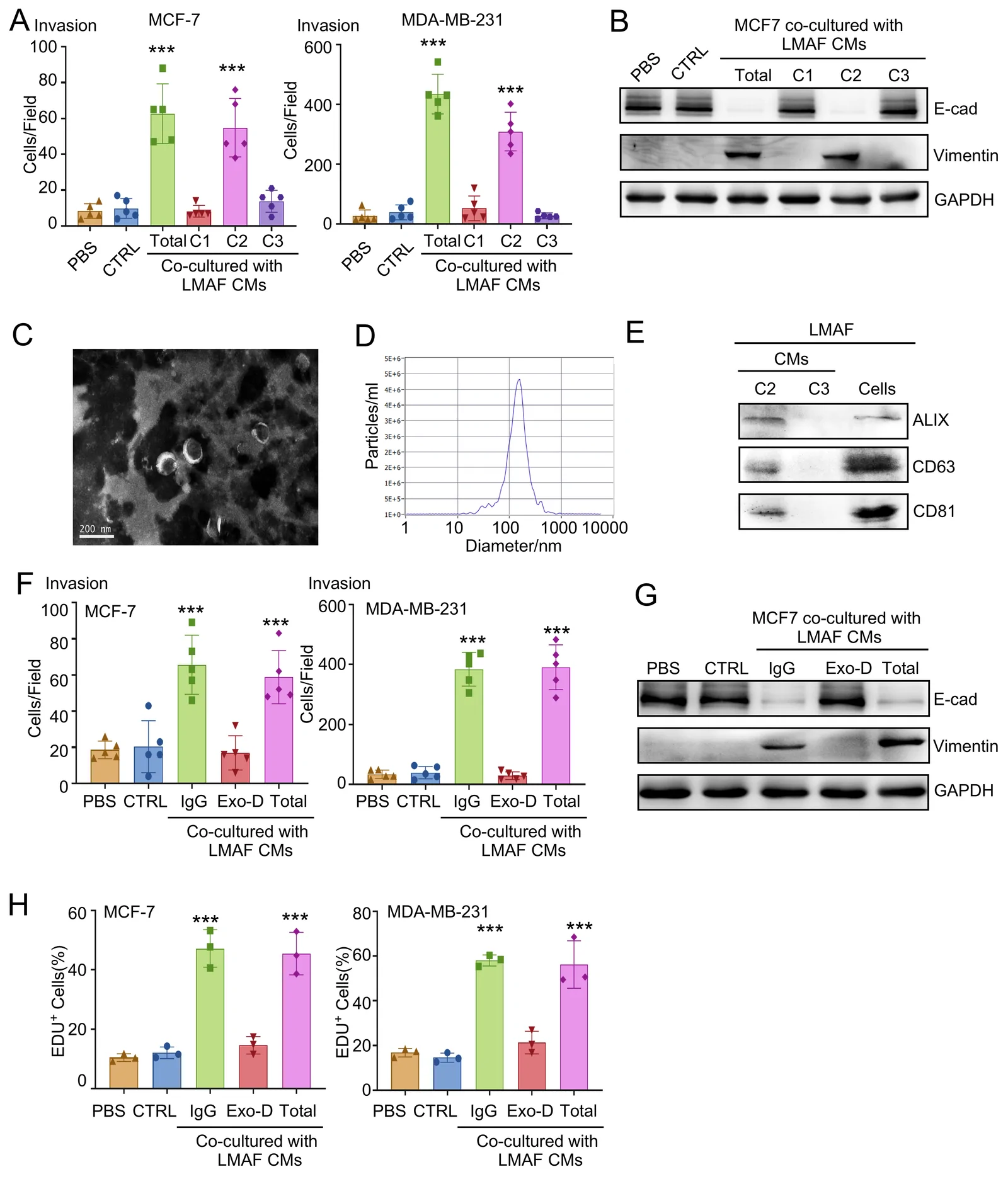
** LMAFs drove breast cancer EMT, invasion and proliferation through exosomes.** (A-B) CAFs were cultured in DMEM supplemented with exosome-depleted FBS. CMs derived from lung metastasis-associated fibroblasts (LMAFs) were fractionated via differential ultracentrifugation. Based on their sedimentation properties, three distinct fractions were isolated and designated as C1, C2, and C3 for subsequent experiments. A. MCF-7 cells were treated with specified LMAF-derived fractions (C1, C2, C3) or total conditioned media (Total). Total CM from CTRL-CAFs (CTRL) and PBS were employed as controls. Quantification of the Transwell invasion assay is shown (*n* = 5 independent patients). B. Representative immunoblotting for E-cadherin (E-cad) and Vimentin of MCF-7 treated with indicated LMAF CM fractions. MCF-7 treated with PBS or CTRL-CAF CMs were used as control (*n* = 3 independent patients). C. Electron-microscopic observation of whole-mounted exosomes purified from LMAFs with the typical morphology and size (40-150 nm). D. Nanoparticle tracking analysis (NTA) of LMAF CM-derived component 2 (*n* = 3 independent patients). E. Representative immunoblotting for Alix, CD63 and CD81 of cell lysates or CM-derived C2 and C3 fractions of LMAFs (*n* = 3 independent patients). The C1 fraction, which contains larger vesicles and cellular debris, was not analyzed because it is not the exosome-enriched fraction. (F-G) Exosomes were removed from LMAF conditioned media (CM) using a commercial isolation kit to generate exosome-depleted CM (Exo-D). Total LMAF CM and IgG treatment served as experimental controls. F. MCF-7 cells (left) and MDA-MB-231 (right) cells were treated with unprocessed LMAF-derived CMs (Total), Exo-D CMs (exosome-depleted CMs) or IgG (IgG-treated CMs). Total CM from CTRL-CAFs (CTRL) and PBS were employed as controls. Quantification of the Transwell invasion assay is shown (*n* = 5). G. Representative immunoblotting for E-cadherin (E-cad) and Vimentin of MCF-7 treated with LMAF-derived CMs, Exo-D CMs or IgG. Total CM from CTRL-CAFs (CTRL) and PBS were employed as controls (n = 3 independent patients). H. EdU incorporation assay in MCF-7 cells (left) and MDA-MB-231 cells (right) treated with LMAF-derived CMs, Exo-D CMs or IgG. Total CM from CTRL-CAFs (CTRL) and PBS were employed as controls (*n* = 3 independent patients). Data are presented as mean ± s.d. *** p < 0.001 by one-way ANOVA with Dunnett's test compared to CTRL group in (A, F, H).

**Figure 3:**
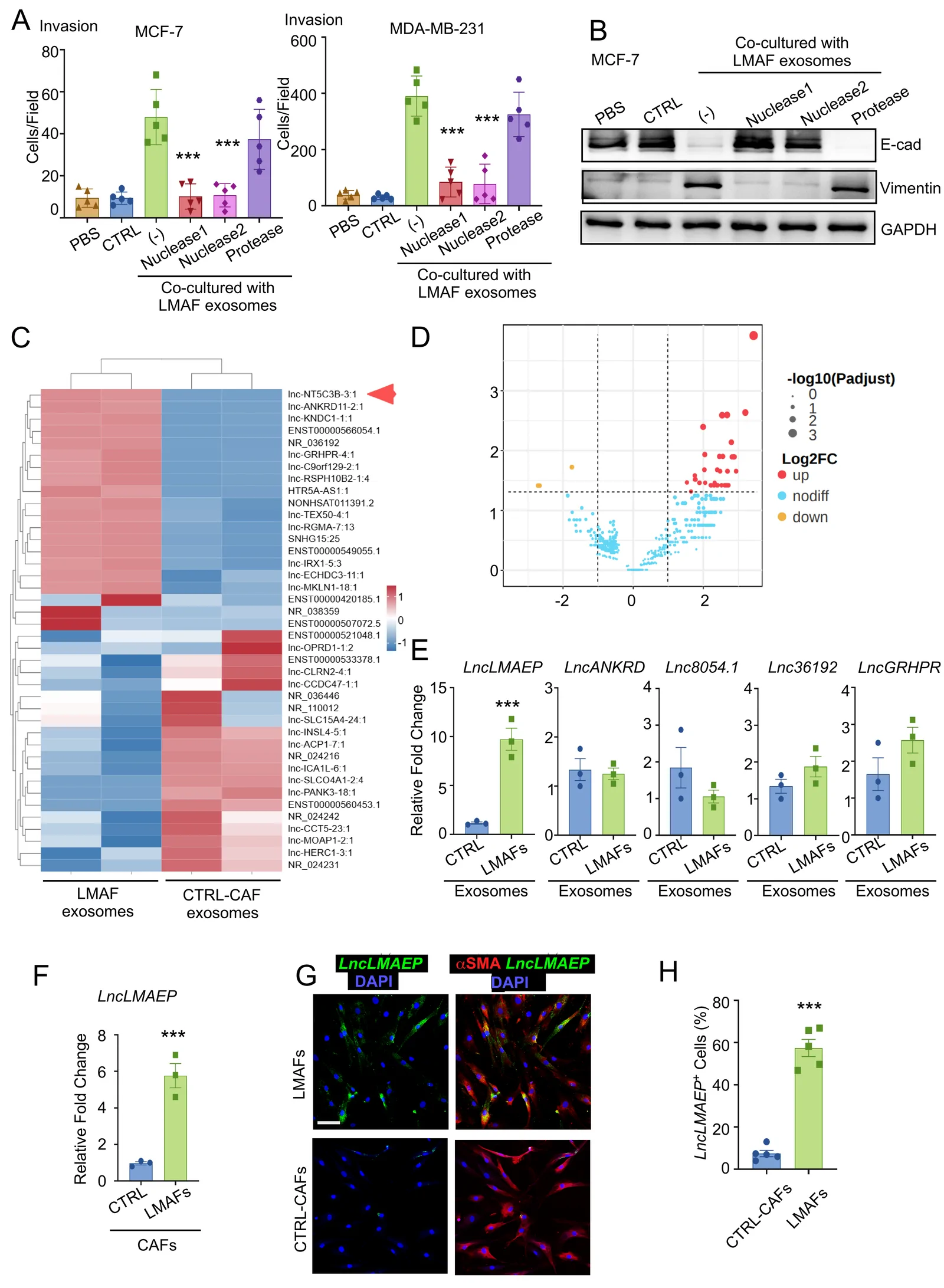
** LMAEP emerged as a signature lncRNA in pro-metastatic exosomes of LMAFs.** (A-E) CAFs were cultured in DMEM supplemented with exosome-depleted FBS. Exosomes were isolated from LMAF CMs via differential ultracentrifugation for further experiments. Exosomes isolated from CTRL-CAFs (CTRL) were employed as control. (A-B) MCF-7 and MDA-MB-231 cells were treated with LMAF-derived exosomes pre-incubated with or without nuclease or protease. Nuclease 1, RNase A; Nuclease 2, RNase I. Protease, Proteinase K. A. Quantification of the Transwell invasion assays evaluating the invasion capacity of MCF-7 (left) and MDA-MB-231 cells (right) with the indicated treatment is shown (*n* = 5). B. Representative immunoblotting for E-cadherin (E-cad) and Vimentin of MCF-7 with the indicated treatments (*n* = 3 independent experiments). C. Heatmap displaying differentially expressed (DE) lncRNAs in LMAFs compared to CTRL-CAFs. Data are presented as log_2_-transformed expression values, with color scales representing row-normalized Z-scores (*n* = 2 biological replicates). D. Volcano plot highlighting DE lncRNAs between LMAFs and CTRL-CAFs. Significantly up-regulated and down-regulated transcripts are indicated by red and blue dots, respectively (log2 FC > 1, p < 0.05). E. qRT-PCR analysis of the expression of several lncRNAs in the exosomes of LMAFs and CTRL-CAFs. Data were normalized to spike-in control and are presented as relative fold change (*n* = 3). F. qRT-PCR analysis of LMAEP expression in LMAFs and CTRL-CAFs. Data were normalized to GAPDH and are presented as relative fold change (*n* = 3). G. Representative images of Fluorescence In Situ Hybridization (FISH) for LMAEP (green) and immunostaining of α-SMA (red) in primary LMAFs and CTRL-CAFs (*n* = 5). Nuclei were counterstained with DAPI (blue). Left panels: overlay of LMAEP (green) and DAPI (blue). Right panels: overlay of LMAEP (green), α-SMA (red), and DAPI (blue). Scale bar, 40 μm. H. Quantitative analysis of LMAEP-expressing CAFs in primary LMAF and non-metastatic CAFs (CTRL-CAFs) (*n* = 5). Data represent the percentage of LMAEP^+^ cells as determined by FISH. Data are presented as mean ± s.d. *** p < 0.001 by one-way ANOVA with Dunnett's test compared to (-) group in (A), *p* values by unpaired Student's t-test in (E, F, H).

**Figure 4:**
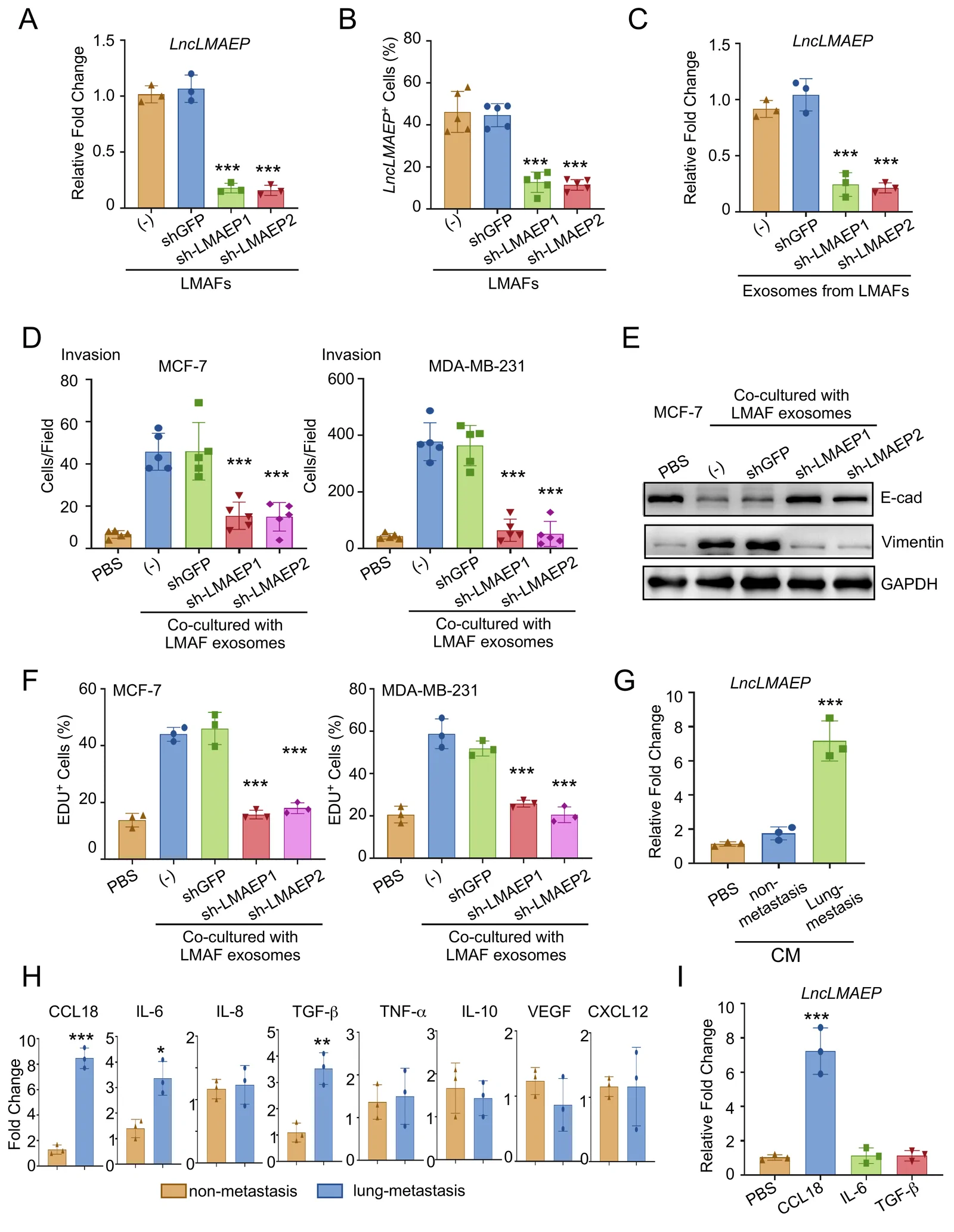
** LMAEP knockdown abrogated LMAF-induced tumor cell invasion and proliferation.** (A-F) LMAFs were transduced with lentiviral shRNAs targeting LMAEP (sh-LMAEP1 and sh-LMAEP2) or a control shRNA (shGFP). Following transduction, cells were maintained in culture medium supplemented with exosome-depleted FBS for subsequent assays. Non-transduced LMAF group (-) was employed as control. A. qRT-PCR analysis of LMAEP expression levels in LMAFs with the indicated treatments. Data were normalized to GAPDH and are presented as relative fold change (*n* = 3). B. Quantitative analysis of LMAEP-expressing CAFs in LMAF populations with the indicated treatments (*n* = 5). Data represent the percentage of LMAEP^+^ cells as determined by FISH. (C-F) C. Exosomes were isolated via differential ultracentrifugation from the CM of LMAFs transduced with either LMAEP-targeting or control shRNAs. CM from un-transduced LMAFs was utilized as an additional baseline control. C. qRT-PCR analysis of LMAEP expression in exosomes harvested from the indicated conditioned media (CM). Data were normalized to spike-in control and are presented as relative fold change (*n* = 3 biological replicates). D. Quantification of the Transwell invasion assays evaluating the invasion capacity of MCF-7 (left) and MDA-MB-231 cells (right) with the indicated treatments is shown (*n* = 5). E. Representative immunoblotting for E-cadherin (E-cad) and Vimentin of MCF-7 with the indicated treatments (*n* = 3 independent experiments). F. EdU incorporation assay in MCF-7 (left) or MDA-MB-231 (right) cells treated with exosomes from LMAFs transduced with shGFP or LMAEP-targeting shRNAs (sh-LMAEP-1 and sh-LMAEP-2). Exosomes from non-transduced LMAFs (-) served as an additional control (*n* = 3). G. qRT-PCR analysis of LMAEP expression in exosomes from normal fibroblasts treated with conditioned media (CM) derived from primary tumors of breast cancer patients with or without lung metastasis, or PBS. Data were normalized to spike-in control and are presented as relative fold change (*n* = 3). H. ELISA analysis of different metastasis-related cytokines in tumor CM from lung-metastatic and non-metastatic patients (*n* = 3). I. qRT-PCR analysis of LMAEP expression in exosomes from normal fibroblasts treated with recombinant CCL18, IL-6, TGF-β or PBS. Data were normalized to spike-in control and are presented as relative fold change (*n* = 3). Data are presented as mean ± s.d. *** p < 0.001 by one-way ANOVA with Dunnett's test compared to shGFP group in (A-D, F), to non-metastasis group in (G), to PBS group in (I). *** p < 0.001 by unpaired Student's t-test in (H).

**Figure 5:**
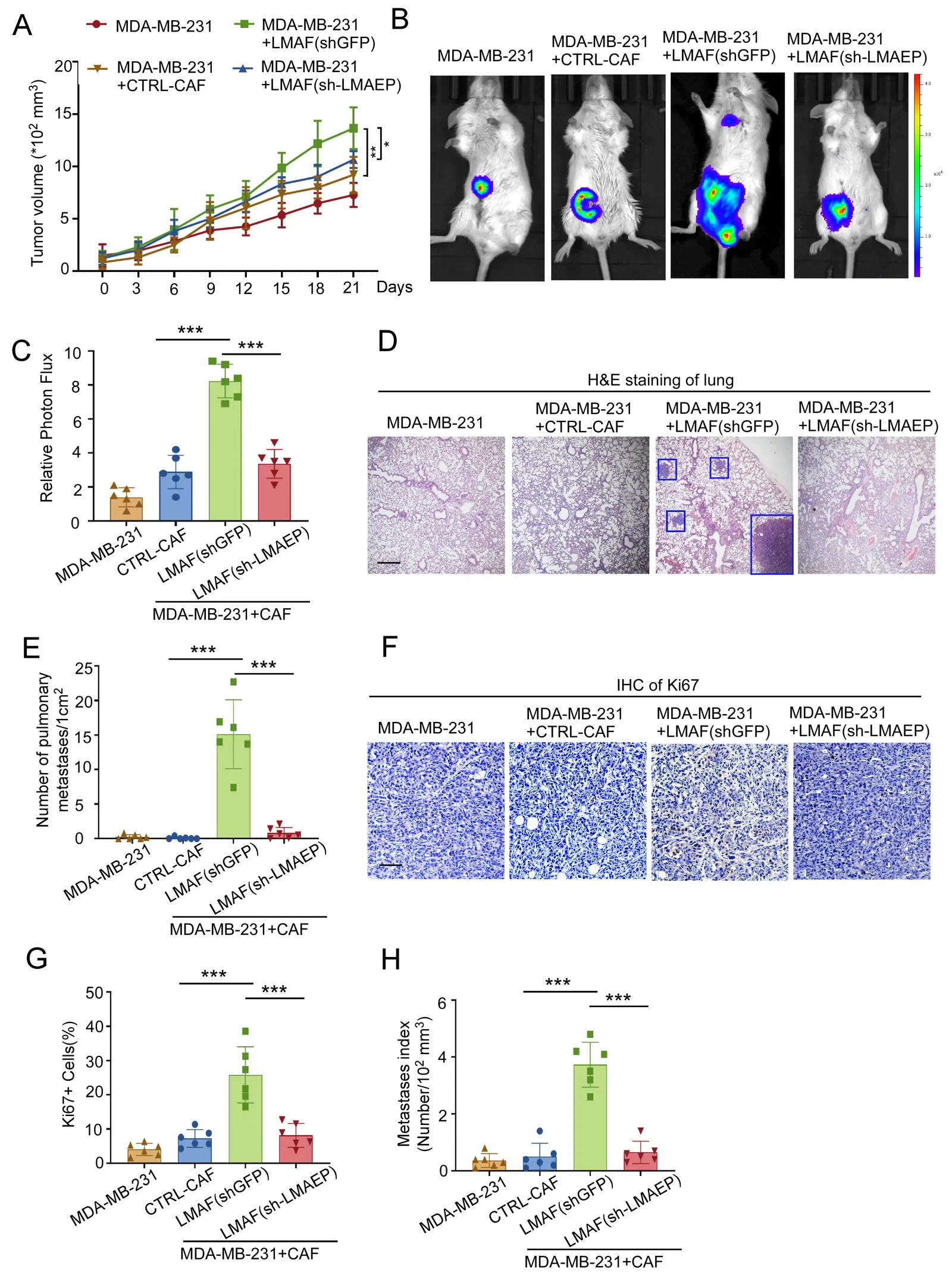
** Targeting LMAEP suppressed LMAF-driven lung metastasis* in vivo.*** (A-D) MDA-MB-231 cells expressing luciferase (1 × 10^6^ per mouse) were co-injected with either CTRL-CAFs, shGFP- or sh-LMAEP-transduced LMAFs into the fourth mammary fat pad of NSG mice. Injection of MDA-MB-231 cells alone served as a negative control. Tumor growth and spontaneous metastasis were monitored every 3 days. A. Tumor growth curves of MDA-MB-231 cells orthotopically co-implanted with the indicated CAFs or injected alone (*n* = 6 mice per group). Tumor volumes were measured every 3 days using calipers. Data are presented as mean ± s.e.m. B. Representative bioluminescence monitoring (IVIS) images showing the distribution and intensity of luciferase-labeled MDA-MB-231 cells in mice from the indicated groups. The color scale represents relative photon flux (photons/s/cm^2^), with red and blue indicating high and low signal intensities, respectively. C. Quantitative analysis of the relative photon flux of IVIS data in (B) for each group. *n* = 6 mice per group. Data were mean ± s.d. D. Representative images of hematoxylin and eosin (H&E) staining for lung metastases in MDA-MB-231 tumor-bearing mice from the indicated groups. Boxes indicate representative metastatic foci. Scale bar, 1 mm. E. Quantification of pulmonary metastatic nodules per 1 cm² of lung tissue in the indicated groups (*n* = 6 mice per group). F-G. Representative images (F) and quantification (G) of Ki67 immunohistochemical staining on primary tumor sections from the indicated groups. MDA-MB-231 cells were co-injected with CTRL-CAFs, shGFP-LMAFs, or sh-LMAEP-LMAFs; MDA-MB-231 cells alone served as control. Scale bar, 100 μm. *n* = 6 mice per group. H. Quantification of metastatic index (number of lung metastatic foci per 10^2^ mm^3^ of primary tumor volume) for each mouse in the indicated groups (*n* = 6 mice per group). * *p* < 0.05, ** p < 0.01, *** p < 0.001 by one-way ANOVA with Tukey test (A, C, E, G, H).

## Data Availability

All data generated in the context of this study is available from the authors upon reasonable request.
